# Artificial Intelligence in Radiology: Transforming Cancer Detection and Diagnosis

**DOI:** 10.7759/cureus.96518

**Published:** 2025-11-10

**Authors:** Shubham Gupta, Ashirwad P, G Harsha Vardhan Reddy, Karthick Natarajan, Vishal Srivastava, Jatin Goda

**Affiliations:** 1 Radiology, University of Jammu, Jammu, IND; 2 Radiology, Asian Institute of Gastroenterology (AIG) Hospitals, Hyderabad, IND; 3 Hepato-Pancreato-Biliary (HPB) Surgery &amp; Liver Transplantation, Institute of Liver and Biliary Sciences, New Delhi, IND; 4 Computer Science and Engineering, University of Madras, Chennai, IND; 5 Marketing, Indian Institute of Management (IIM) Kashipur, Kashipur, IND; 6 Anatomy, Dr. N. D. Desai Faculty of Medical Science and Research, Dharmsinh Desai University, Nadiad, IND

**Keywords:** artificial intelligence, cancer detection, deep learning, medical imaging, radiogenomics

## Abstract

Artificial intelligence (AI) is increasingly integral to radiological oncology, where it supports complex image interpretation, quantitative tumor analysis, and clinical decision-making. This review synthesizes state-of-the-art developments in AI applications across major cancer types: breast, lung, prostate, brain, gastrointestinal, and metastatic disease, focusing on deep learning, radiomics, and radiogenomics frameworks. These technologies have demonstrated substantial improvements in lesion detection, segmentation accuracy, risk prediction, and molecular phenotype inference, with performance metrics that approach or surpass those of experienced radiologists. The review also explores AI integration with diverse imaging modalities, including computed tomography (CT), magnetic resonance imaging (MRI), positron emission tomography/computed tomography (PET/CT), and digital mammography, while examining AI’s impact on workflow triage, report standardization, and radiologist efficiency. Despite these advancements, key challenges persist, including limited model generalizability across populations and institutions, data silos, regulatory uncertainty, and the need for explainable AI outputs in clinical contexts. Emphasis is placed on enabling strategies such as federated learning, multicenter data harmonization, post-deployment monitoring, and integration into picture archiving and communication system/radiology information system (PACS/RIS) infrastructure. The article provides a critical evaluation of the current landscape while outlining strategic directions for safe, equitable, and effective implementation. Rather than replacing radiologists, AI is emerging as a collaborative partner shaping a future of data-driven, personalized oncologic imaging that aligns with precision medicine goals.

## Introduction and background

Since the invention of X-rays, radiology has developed tremendously, with the simple two-dimensional radiology giving way to advanced multimodal imaging systems like magnetic resonance imaging (MRI), computed tomography (CT), positron emission tomography (PET), and integrated systems like PET-CT and PET-MRI [1]. Earlier applications of artificial intelligence (AI) in radiology included the use of AI in PET imaging for brain imaging to help diagnose conditions like dementia and nuclear myocardial perfusion scans, which have used AI for decades to aid in diagnosing inducible ischemia. The inventions have essentially transformed oncological practice by making it possible to visualize, stage, monitor treatment, and surveil metastasis in a non-invasive manner [1]. As the world grapples with the ever-rising cancer burden, the need to access advanced imaging services is also on the rise, which is putting a lot of pressure on health systems and radiology departments [2]. The growing volume of cancer imaging data, driven by the increasing incidence of cancer worldwide, presents a significant challenge to healthcare systems. This underscores the urgent need for AI adoption to manage the data load and enhance diagnostic capabilities while improving efficiency in radiology departments.

Irrespective of these technological innovations, traditional radiological interpretation is still limited by various limitations. Radiologists are faced with time limitations to review large, complex datasets that can result in interpretive inconsistency, diagnostic delay, and error due to fatigue [3]. There are inter-observer differences, particularly in less severe or ambiguous results, and this can undermine the accuracy of the diagnosis. Also, the increased application of imaging in cancer screening and monitoring programs and regimes has led to the increasing workload that cannot be handled by the number of available specialists [4].

**Figure 1:**
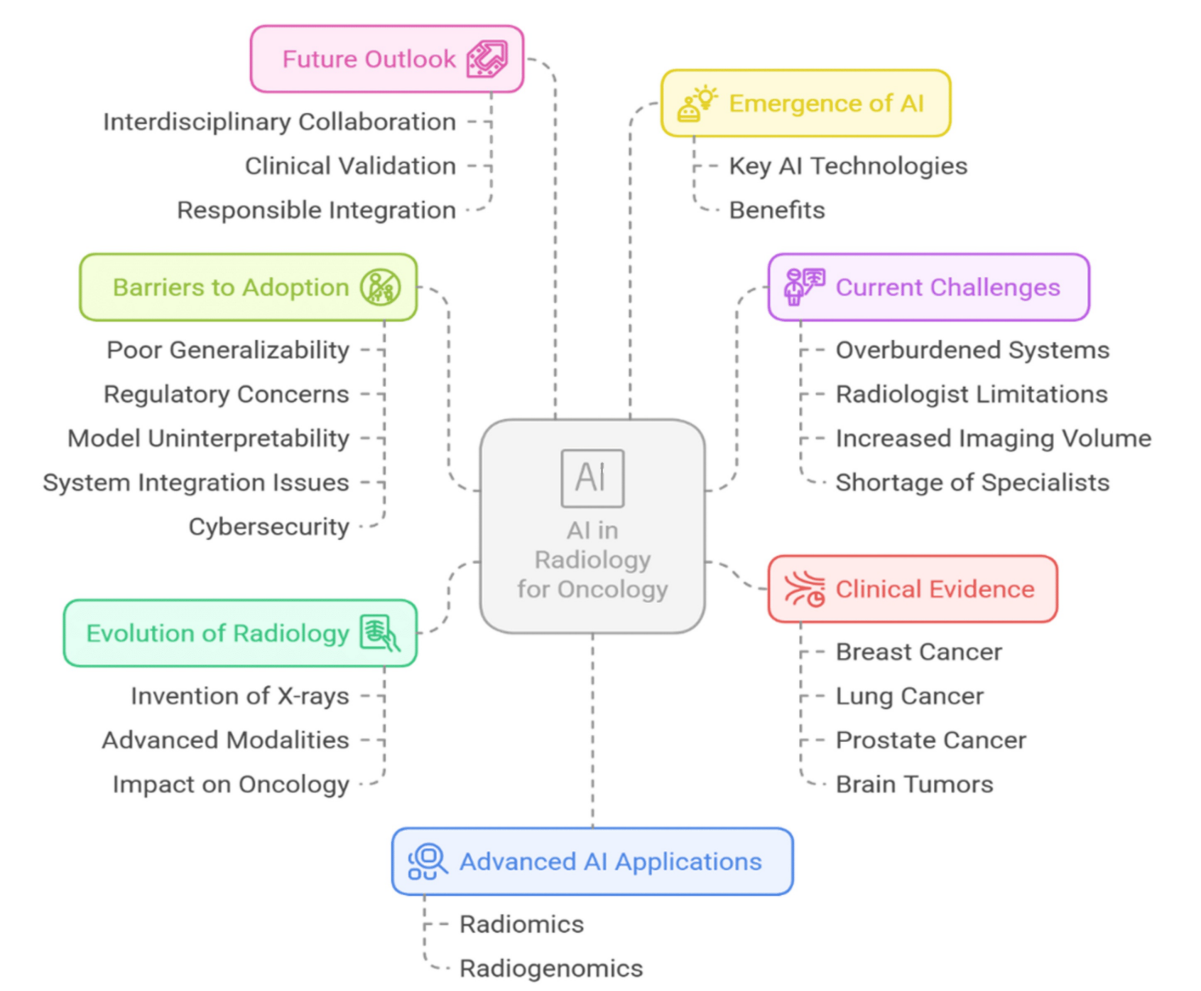
Mind map of AI in radiology for oncology: evolution, challenges, and future directions AI: artificial intelligence Image credits: Shubham Gupta

AI, a field of computer science focused on creating systems that can simulate human intelligence, has emerged as a promising approach to addressing these challenges. AI encompasses a variety of models, including machine learning (ML) and deep learning (DL), which are specifically designed to perform tasks such as image analysis, decision-making, and pattern recognition. In the last 10 years, AI, especially AI that uses ML and DL, has shown incredible promise in automating image analysis, assisting in diagnostic decision-making, and making radiologists more efficient. Several companies, including IBM Watson Health, Google DeepMind, and Siemens Healthineers, have made significant strides in developing AI tools for radiology, focusing on improving diagnostic accuracy and workflow efficiency [5]. DL is a branch of ML based on multilayered neural networks (e.g., convolutional neural networks (CNNs)) that learn hierarchical features in the data in an unsupervised manner without direct feature engineering [6]. These abilities are especially appropriate to the complicated visual activities of the radiological workflows. Figure 1 shows a conceptual overview of AI's role in oncologic radiology, encompassing its evolution, clinical applications, current challenges, and adoption barriers.

Radiomics only adds to the role of AI in oncology. Radiomics is the extraction of high-throughput quantitative features from medical images that can reveal patterns and biomarkers that are not visible to human observers [7]. Radiomic features have the potential to provide tumor phenotype, prognosis, and treatment response information when combined with clinical and genomic data, as these features reveal underlying patterns in medical images that reflect the biological properties of tumors. These insights can be used to predict how tumors might respond to specific treatments or evolve and aid in the creation of precision oncology [8]. The related discipline of radiogenomics seeks to correlate the imaging phenotype and the genetic one by linking the visual patterns observed in radiological images with the genetic and molecular characteristics of the tumor, such as specific mutations or gene expression profiles. This approach enables a more comprehensive understanding of the tumor's biology and behavior, which can aid in better diagnosis and prognosis.

AI has already demonstrated effectiveness in various types of cancer in clinical practice, with earlier AI applications emerging in the 1990s, such as expert systems for tumor detection, and more advanced models, including DL, gaining prominence in the 2000s and 2010s [5]. The DL algorithms have been used to detect malignancies on mammograms with sensitivity comparable to or even better than that of expert radiologists in breast cancer screening, resulting in decreased recall rates and unnecessary biopsies [9]. In the case of lung cancer, AI-based processing of low-dose CT (LDCT) images can enhance early diagnosis of pulmonary nodules and stratify the risk of the disease according to the nodule features [10]. In prostate cancer, the AI-assisted interpretation of multiparametric MRI (mpMRI) allows the correct identification of lesions, and the standardized scoring systems, such as Prostate Imaging Reporting and Data System (PI-RADS), increase inter-reader reliability [11]. AI models have been realized in neuro-oncology to automate tumor segmentation, histologic grading, and molecular subtype prediction based on MRI data to support clinical decision-making and planning [12].

Although these advancements demonstrate the revolutionary power of AI, there are still some obstacles to its mass acceptance. One of the issues is the ability of AI models to generalize to different clinical environments, as most are trained either on small or institution-specific data. Moreover, clinical implementation is complex due to regulatory uncertainty, uninterpretability, ethical issues, and integration issues with pre-existing hospital information systems. Cybersecurity risks, especially with large datasets of imaging data being stored in or processed in cloud settings, are also to be taken into consideration. Nevertheless, persistent interdisciplinary cooperation and a strong clinical validation are likely to lead to the responsible use of AI in everyday oncological imaging.

Objective of the review

This review aims to provide a comprehensive synthesis of current AI applications in radiology for cancer detection and diagnosis. It has explored technological foundations, evaluated performance across major cancer types, examined the integration of AI with imaging modalities, compared the feasibility and cost-effectiveness of different AI models in relation to radiologists, highlighted existing limitations, and proposed directions for future research and clinical translation.

## Review

Methodological considerations

The review is based on a comprehensive analysis of peer-reviewed literature published between January 2015 and June 2025. Articles were identified through searches in PubMed, Scopus, and IEEE Xplore using the search terms "AI in radiology," "cancer imaging," "radiomics," "deep learning," and "oncology diagnosis." Only original research, reviews, and systematic reviews that focused on the use of AI-based tools for cancer imaging were included in this review. Priority was given to studies that reported clinical validation or those that utilized large datasets from different cancer types and imaging modalities.

Studies were excluded if they were preclinical, focused on non-imaging-based AI applications, or lacked transparency in their methodologies. Additionally, articles were excluded if they did not directly relate to the integration of AI in oncologic imaging or if they did not meet the criteria for clinical relevance, such as lacking substantial datasets or proper validation of AI models.

To ensure transparency and replicability, the quantitative data (e.g., area under the curve (AUC), sensitivity, and specificity) were summarized using means and ranges where applicable, and variations in results were qualitatively discussed. Although formal statistical tests for heterogeneity were not performed, qualitative differences in performance metrics across studies, such as variations in AUC values, were considered and discussed to provide a comprehensive view of AI's effectiveness in oncologic imaging. This approach ensured that the review is grounded in high-quality, clinically validated research, while also emphasizing studies that address the integration of AI into practical imaging workflows for cancer diagnosis and treatment.

Foundations of AI in radiology

Radiology AI is based on concepts of computational methods through which machines can learn data patterns [13]. These systems are based on two main paradigms: supervised and unsupervised learning. Supervised learning involves the use of labeled data to learn a mapping of input images to a fixed set of diagnostic labels, and unsupervised learning identifies underlying structure in unlabeled data, enabling applications including clustering and detecting anomalous datasets [14]. Radiology has been transformed by DL, a type of ML that allows algorithms to learn hierarchical representations on their own, based on complex image data [15]. The prevailing DL architecture in image-related applications is CNNs, which are multilayered networks that automatically learn spatial hierarchies, hence particularly useful to detect anatomical structures and pathological changes in radiological images [16]. In contrast to traditional computer-aided detection (CAD) systems, CNNs do not have to be hand-crafted, so there is increased scalability and generalizability of CNNs to other diagnostic tasks [7].

The use of AI with significant imaging techniques like CT, MRI, PET, and digital mammography has widened the range of possibilities in diagnosis [17]. As an example, CNNs trained on CT images in the Lung Image Database Consortium Image Collection (LIDC-IDRI) have performed with an AUC higher than 0.94 in the classification of pulmonary nodules [18]. On the same note, AI models used in mpMRI have been proven to be more than 90% accurate in the segmentation of prostate lesions to help in the clinical interpretation and decision-making. Radiomics extraction is used to retrieve large amounts of quantitative features of a medical image, including texture, shape, intensity, and wavelet transformations [19]. Such characteristics may indicate tumor heterogeneity, vascularity, and cellular density, which are usually linked to treatment response or prognosis [20]. Radiogenomics goes a step further and matches imaging phenotypes with molecular and genetic profiles. As another example, CT-derived radiomic features have been linked to the epidermal growth factor receptor (EGFR) mutation status in non-small-cell lung cancer (NSCLC), offering a non-invasive method to infer the genome [2].

Even though positive outcomes are observed, the implementation of AI in radiology is not easy in the real world. Models tend to perform poorly when transferred to fresh data because of imaging protocols, patient demographics, and scanner equipment. The growing use of collaborative projects such as the Medical Imaging Data Resource Center (MIDRC) and publicly available benchmarks such as The Cancer Imaging Archive (TCIA) is working to achieve standardization of datasets and support external validation [11]. Besides, clinical and regulatory norms are being formed to guarantee the reproducibility and safety of AI-aided diagnostics.

AI in breast cancer imaging

Mammography is the most common screening test used in the detection of breast cancer, as it is the most common cancer occurring in women. Its diagnostic sensitivity is, though, limited by interpretive variability, particularly in women with dense breasts, which results in false positives and false negatives of malignancies [21]. AI has demonstrated transformative potential in enhancing mammographic interpretation. Large-scale DL models, e.g., trained on the Digital Mammography DREAM Challenge dataset or the UK OPTIMAM dataset, have demonstrated sensitivity and specificity at the expert radiologist level [6]. As an example, an article published in Nature stated that on US datasets, the Google AI system (Google, Mountain View, CA, US) decreased false positives by 5.7% and false negatives by 9.4%, and had the same pattern in UK datasets [22].

AI-based tools ProFound AI (iCAD, Nashua, NH, US) and Transpara (ScreenPoint Medical, Nijmegen, The Netherlands) have already been cleared by the FDA and are incorporated into clinical practice. These systems show risk scores, lesion detection heatmaps, and triage, all of which are decision support to radiologists. It has been demonstrated in clinical trials that ProFound AI enhances the radiologist's sensitivity by 8% and reduces the reading time by 52% [23]. Besides detection, risk stratification is being applied with the help of AI. Composite tools that incorporate mammographic density, lesion morphology, and patient history may be used to provide individual risk scores to inform the decision to use supplemental imaging, including ultrasound or MRI [10]. In one multicenter trial, an AI-based risk model demonstrated a higher AUC of 0.71 than the Tyrer-Cuzick model at 0.61 in predicting the five-year risk of breast cancer [24].

**Figure 2:**
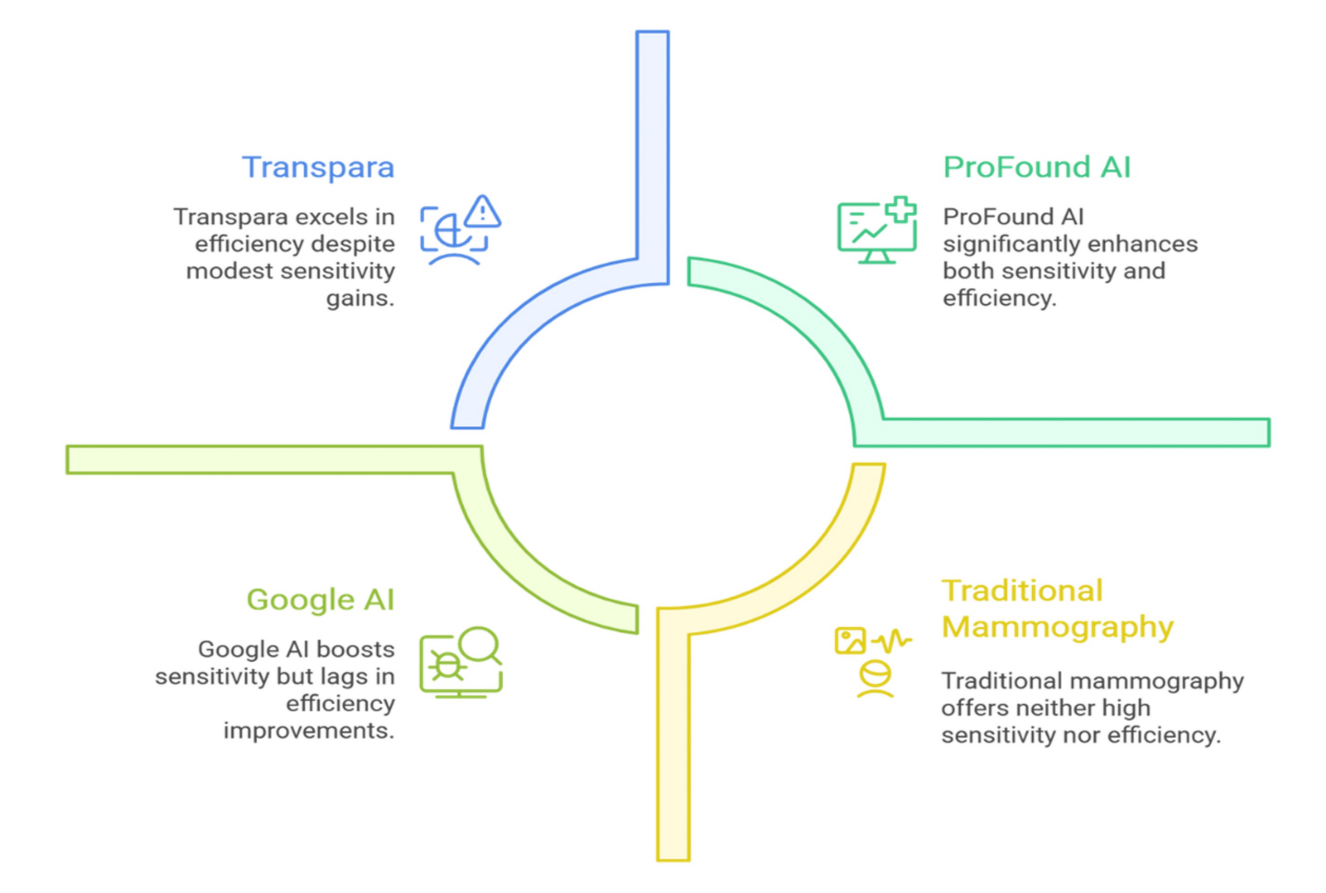
Overview of AI-based solutions and traditional approaches in mammographic breast cancer screening AI: artificial intelligence Image credit: Shubham Gupta

AI also addresses workforce challenges by reducing the workload. When there are large-scale screening programs, AI can be used to prescreen the cases that are low-risk, and radiologists can concentrate only on the scans that are complex or ambiguous. The most recent study has shown that AI prescreening, on a sample of more than 250,000 mammograms, has allowed reducing the workload of radiologists by 44.5% without compromising the accuracy of the diagnosis [25]. Nonetheless, limitations remain. The performance of many AI models degrades on out-of-distribution data, such as images of underrepresented ethnicities or analog systems that are older. It is also feared that automation bias could occur, in which radiologists tend to overly depend on AI results, which could fail to capture discordant cases [17]. Such approaches as explainable AI (XAI), saliency maps, and attention mechanisms attempt to enhance transparency and trust by presenting features that the model took into account when making a decision [26]. Figure 2 illustrates how various AI tools and traditional mammography differ in sensitivity and efficiency.

Lung cancer detection and diagnosis

Lung cancer causes the greatest mortality rate of all cancer cases in the world, and this is mainly because lung cancer is diagnosed late. LDCT screening saves lives; however, the screening faces logistic and diagnostic issues because of the large scan volume and reader performance variability [12]. The systems based on AI have been created to automate the process of detecting nodules and determining their risk. CNNs that have been trained on the National Lung Screening Trial (NLST) dataset and the LIDC-IDRI repository have demonstrated AUCs of 0.87 to 0.94 in differentiating benign and malignant nodules [25]. These models consider the size of the nodule, spiculation, volume doubling time, and the contextual lung characteristics. As an example, the Virtual Nodule Clinic provided by Optellum is based on AI to stratify nodules according to the risk of malignancy and is FDA-cleared to be used in clinical triage applications [27].

AI is also valuable in post-detection workflows. It can track the nodules' development through serial scans with 3D volumetric evaluation, which has less interobserver differences and is able to better assess the growth patterns. This aids in the decision to do a biopsy, surgery, or surveillance in clinical practice [5]. With the help of AI, there is a better triaging process since suspicious scans are marked to be looked at faster. One of the studies showed that prioritizing LDCT scans with high-risk nodules based on AI led to reduced diagnostic intervals by 15% and earlier management of stage I cancers [17]. The same applies especially in a scenario where there is a low availability of radiologists.

In addition to detecting, radiomics has allowed the extraction of predictive imaging biomarkers of tumor aggressiveness and response to treatment. CT and PET image quantitative features entropy, skewness, and heterogeneity have been linked to survival and resistance to treatment in NSCLC [22]. Radiogenomic analyses have also linked the imaging radiographic characteristics with gene mutation, including KRAS and ALK, and this could be used in personalizing the treatment approaches [19]. With the promise comes the need for strong external validation and regulatory control to implement in the real world. Variability in slice thickness, reconstruction algorithm, and contrast may influence AI performance. Future studies like the Pathology Nodules Optics (PANOPTIC) and LUNGx are being planned to determine how generalizable models will be across institutions and equipment vendors [3].

Prostate cancer and AI-based MRI analysis

Prostate cancer diagnosis is particularly complex due to multifocal growth patterns and heterogeneous lesion presentation. While mpMRI has significantly enhanced detection capabilities, interpretation remains variable across radiologists, especially in equivocal PI-RADS 3 lesions [28]. AI models trained on datasets such as ProstateX have shown high diagnostic performance, offering more consistent evaluation across centers and clinicians [29].

Furthermore, AI integration in biopsy workflows, particularly in MRI/ultrasound fusion-guided systems, has yielded clinically significant improvements. Platforms like Artemis have demonstrated increased detection rates of aggressive tumors, especially in hard-to-reach anterior and apical zones [30]. These improvements extend to radiotherapy planning, where AI tools now support automated prostate segmentation, expediting dose planning and increasing precision [31].

To facilitate clinical trust and regulatory adoption, XAI methods such as Grad-CAM and saliency maps have been introduced, highlighting features that influence model predictions [20]. Ethical integration also demands cross-validation on diverse imaging platforms and populations to prevent performance bias and enhance generalizability [32]. These advances suggest that AI, when responsibly implemented, will significantly improve accuracy, efficiency, and equity in prostate cancer imaging and treatment. Table 1 shows the multifaceted applications of AI in prostate cancer imaging and biopsy, highlighting its impact on diagnostic accuracy, biopsy precision, treatment planning, and clinical integration.

**Table 1:** Role of artificial intelligence in prostate cancer imaging and biopsy AI: artificial intelligence; mpMRI: multiparametric magnetic resonance imaging; PI-RADS: Prostate Imaging Reporting and Data System; CNN: convolutional neural network; AUC: area under the curve; MRI: magnetic resonance imaging

Clinical domain	AI contribution	Clinical impact	AI tools/methods	References
mpMRI Interpretation	Reduces variability, enhances lesion detection	More accurate diagnosis, especially in PI-RADS 3 cases	CNNs trained on ProstateX dataset; AUC > 0.90	Mun et al. [4]
Lesion Classification	Automated PI-RADS scoring and zone-based lesion mapping	Supports radiologists with varying experience	Deep learning-based segmentation and classification	Langlotz et al. [29]
Biopsy Targeting	AI-enhanced MRI/ultrasound fusion improves lesion localization	15% higher detection of clinically significant tumors	Fusion biopsy systems with AI targeting (e.g., Artemis)	Kumar et al. [30]
Radiotherapy Planning	Automated volumetric segmentation	Enables precise radiation dosing and planning	bkFusion, volumetric AI models	Khalifa and Albadawy [11]
Explainability & Trust	Visual explanation of model decisions	Enhances clinician confidence in AI-assisted interpretations	Grad-CAM, saliency maps (Explainable AI)	Harmon et al. [20]
Ethical & Technical Integration	Emphasizes fairness, cross-validation, and workflow incorporation	Promotes generalizability and safe clinical deployment	Validation across platforms, scanners, and ethnic populations	Huang et al. [16]

Brain tumor segmentation and classification

AI in Imaging Modalities: MRI in Brain Tumor Imaging

Imaging plays a crucial role in neuro-oncology in the diagnosis and follow-up of brain tumors (particularly gliomas). The most aggressive one, glioblastoma multiforme (GBM), requires accurate demarcation and classification to be managed. MRI is the most preferred modality because it is multifaceted in T1, T2, fluid-attenuated inversion recovery (FLAIR), and diffusion-weighted imaging (DWI) sequences [32].

AI in Organ-Specific Oncology Applications: Brain Tumors

Segmentation algorithms are very successful in automating the process of demarcating tumor subregions based on AI. Architectures that are CNN-based, e.g., U-Net and DenseNet, have achieved Dice similarity coefficients of over 0.90 in BraTS benchmark challenges, comparable to manual expert segmentation [24]. Such models simplify the process of radiotherapy planning and volumetric tracking in a short time.

Tumor classification is another area where AI models excel. Trained algorithms on mpMRI can distinguish high-grade gliomas from low-grade gliomas with classification percentages of over 85% [33]. Inputs to such models are radiomic features such as entropy, skewness, and intensity gradients. AI is also advancing non-invasive molecular profiling. A number of studies have demonstrated that CNN-based radiogenomic models can predict isocitrate dehydrogenase (IDH) status mutation and O6-methylguanine-DNA methyltransferase (MGMT) promoter status methylation with sensitivities of up to 80%, eliminating the need to biopsy inpatients with inoperable tumors or risk of surgery [15]. These are important markers in identifying the response to chemotherapy and prognosis.

Ethical, Regulatory, and Practical Challenges in Neuro-Oncology

Additional advantages have been based on the incorporation of MR spectroscopy into AI pipelines. Features based on spectroscopy, e.g., choline/N-acetyl-aspartate (NAA) ratios, in combination with perfusion imaging, can be used to differentiate between tumor progression and treatment effects such as pseudoprogression [34]. Such multimodal inputs become more confident in diagnosis by AI systems trained on them in ambiguous follow-up scans. However, generalizability remains a hurdle. The performance of models may become worse between imaging protocols, field strengths, or scanners. TCIA and federated learning projects are encouraging data standardization and external validation, multi-institutional projects [35]. The use of explainability is particularly problematic in the field of neuro-oncology, where the predictions may affect high-risk surgical decisions. Uncertainty quantification and saliency-based visualization are under development to convey model confidence to clinicians [36]. With more robust and interpretable models, AI holds the potential to help neurosurgeons and oncologists personalize treatment and minimize the delay in diagnosis.

Gastrointestinal cancers

AI in Imaging Modalities: CT, MRI, and PET/CT

Gastrointestinal (GI) malignancies (colorectal, gastric, hepatic, and pancreatic cancer) are problematic in terms of diagnosis and logistics because they have insidious presentations in the early stages of the disease and are anatomically complex. GI imaging is also incorporating AI tools to enhance early diagnosis, decrease variability, and assist with treatment planning [37]. In CRC screening, AI models on CT colonography have achieved polyp sensitivity of 93%-100% for polyps 6 mm or larger with per-polyp detection rates of more than 93% and false-positive rates that are substantially lower than conventional CAD systems [36]. Some of the tasks that are automated in these models include segmentation of the colon and detection of flat lesions that are important in mass screening.

AI in Organ-Specific Oncology Applications: GI Cancers

The use of AI-based computer-aided detection (CADe) and diagnosis (CADx) systems is changing real-time lesion evaluation in endoscopic applications. CAD software, incorporated into trade platforms such as Olympus ENDO-AID and Fujifilm CAD EYE, points out areas of concern in the course of colonoscopy. In the meantime, CADx systems are trying to differentiate the lesions according to the visual texture and color to distinguish adenomas and hyperplastic polyps [12]. In a Japanese multicenter trial, AI-enhanced colonoscopy was found to increase adenoma detection rates by 15% and per-lesion sensitivity to 93% [18]. Nevertheless, in CADe systems, the risk of over-sensitivity can be high and falsely positive, whereas CADx models are frequently trained on small datasets of histological data, which is quite problematic regarding generalizability [38].

Ethical, Regulatory, and Practical Challenges

The AI-based tools in hepatobiliary oncology are aimed at describing focal liver lesions with the help of radiomic characteristics, including homogeneity, entropy, and edge sharpness. AUCs of more than 0.85 have been reported to differentiate hepatocellular carcinoma (HCC) and benign hepatic nodules [23]. The radiomic analysis is also applicable to predict the microvascular invasion and the risk of recurrence after resection. Radiogenomic models in liver and colorectal metastases have been able to correlate imaging findings with gene mutations, including TP53 and CTNNB1, to stratify patients to receive targeted therapy and immunotherapy [39]. These developments represent a shift toward non-invasive molecular phenotyping.

Nonetheless, challenges persist. AI performance can be decreased by motion artifacts, inconsistent bowel preparation, and variability in the quality of imaging. The ethical issues are automation bias, the absence of transparency in real-time predictions, and the disparity in performance in the underrepresented group. To counter these, confidence levels, heatmap overlay, and alert systems are being incorporated by the developers in order to enhance interpretability. Regulatory agencies such as the FDA and CE are also putting more scrutiny on real-time GI AI tools, and post-market surveillance, as well as clinical trial data, is required [10]. As the generalizability, fairness, and real-life testing continue to improve, AI will likely improve GI cancer diagnostics and increase the screening results around the world. Table 2 shows how AI technologies are being applied across various GI cancer domains, improving diagnostic sensitivity, lesion classification, and molecular targeting while also highlighting implementation challenges and regulatory considerations.

**Table 2:** Role of artificial intelligence in gastrointestinal (GI) cancer imaging and diagnosis CT: computed tomography; AI: artificial intelligence; CAD: computer-aided detection; ML: machine learning; HCC: hepatocellular carcinoma; AUC: area under the curve

Clinical area	AI contribution	Clinical outcome	Tools	References
Colorectal Cancer (CRC)	Detection of polyps and flat lesions on CT colonography	Polyp sensitivity of 93%–100% for ≥6 mm; lower false-positive rates	AI-enhanced CAD systems; colon segmentation algorithms	Rompianesi et al. [21]
Endoscopy–CADe Systems	Real-time identification of suspicious lesions during colonoscopy	↑ adenoma detection rate by 15%; ↑ per-lesion sensitivity to 93%	Olympus ENDO-AID, Fujifilm CAD EYE	Iqbal et al. [18]
Endoscopy–CADx Systems	Differentiation of lesion types (adenoma vs. hyperplastic polyps)	Improved lesion classification during procedures	Texture/color-based ML models	Hosny et al. [6]
Liver Cancer (HCC)	Characterization of focal liver lesions via radiomics	AUC > 0.85 for distinguishing HCC from benign nodules	Radiomic analysis (homogeneity, entropy, edge sharpness)	Santos et al. [23]
Post-surgical Prognosis	Prediction of microvascular invasion and recurrence risk	Supports treatment planning and surveillance	Quantitative image feature extraction	Hunter et al. [3]
Radiogenomics	Linking imaging phenotypes to gene mutations (e.g., TP53, CTNNB1)	Enables personalized immunotherapy and targeted therapy selection	AI-integrated radiogenomic models	Chen et al. [13]
General Limitations	Challenges with generalizability, motion artifacts, and data variability	Potential performance drop in real-world settings	Limited histologic training sets; inconsistent bowel prep	Dangi et al. [10]
Ethical & Regulatory	Addressing automation bias and transparency in predictions	Enhanced model trust, fairness, and accountability	Confidence overlays, heatmaps, alert systems, and FDA/CE oversight	Hosny et al. [6]

Whole-body oncology and metastasis detection

AI has proved to be of great value in the diagnosis and evaluation of metastatic illness in different organ systems. With whole-body oncologic imaging, especially PET/CT, AI models can help detect small metastases in bones, lungs, liver, and lymph nodes, where anatomical or physiological uptake overlap can provide misleading information to human viewers [37]. Such tools are particularly useful in cancers that have a high tendency to spread, including breast, prostate, and melanoma. CNNs trained with whole-body PET/CT data have shown an enhanced level of accuracy compared to the traditional CAD systems in both lymph node and skeletal metastases detection, particularly in prostate cancer [14]. As an example, an AUC of a DL model created by Wang et al. was greater than 0.90 in localizing pelvic lymph node metastases, surpassing radiologists [5]. Likewise, AI has demonstrated good results in calculating total lesion glycolysis and metabolic tumor burden in breast cancer and melanoma to monitor response in the course of chemotherapy or immunotherapy [40].

The most important benefit of AI systems is pattern recognition at the level of various anatomical compartments [41]. In contrast to the conventional workflow, where cases are divided by organ or specialty, AI allows performing a multiregion analysis of the suspected uptake sites, automatically reporting suspicious uptake sites, as well as the standard uptake value (SUV) measures, and correlating with anatomical localizations [38]. By so doing, AI diminishes supervision, improves staging quality, and shortens report turnaround time [42]. Besides, firms like Subtle Medical and AI-Rad Companion by Siemens are combining AI-based tools with picture archiving and communication system (PACS), which auto-fill structured reports with findings, SUV measures, and the evaluation of change over time [28]. AI has also used temporal analytics to identify new metastases or track regression of lesions using serial scans, in lung and colorectal cancer [19].

The transition to whole-body AI modeling is associated with the transition to the development of comprehensive cancer staging tools instead of task-specific algorithms [43]. With the changing models, it is possible that radiogenomic overlays can enable predictions of the likelihood of metastasis on the basis of molecular subtype and site-specific patterns, and thus enhance prognostication and selection of individual therapy [20].

Integration of AI with radiomics and genomics

The combination of AI, radiomics, and genomics is one of the potent areas in personalized oncology. Radiomics is the extraction of high-dimensional imaging features that encode phenotypic heterogeneity, including tumor texture, intensity, and shape [2]. The descriptors are quantitative and give information on the biology, aggressiveness, and microenvironmental conditions of the tumor, much of which is not visible to human eyes. Next-generation sequencing makes it possible to profile driver mutations, transcriptomic signatures, and immune microenvironments in detail with genomics. The intersection of these two data areas-which is made possible by AI-has resulted in radiogenomic models that can predict molecular markers and response to treatment with high accuracy [22].

Radiomics and MRI data have been used in DL models in glioblastoma to predict IDH mutation and methylation of MGMT promoter with an accuracy of over 85% as a non-invasive alternative to biopsy of surgically inaccessible tumors [39]. Aerts et al. have shown the ability of CT-derived texture features to predict EGFR mutations and survival of patients with NSCLC, thereby connecting radiomic phenotypes to actionable genotypes [34]. Breast cancer multi-omics AI models have been used to predict HER2 status using MRI and mammography, and in colorectal cancer to predict microsatellite instability and mismatch repair deficiency (which are both important in immunotherapy decisions) [44]. The provided applications explain how AI-based integration can assist in choosing therapy, reducing invasive diagnostics, and risk stratification of patients [45].

**Figure 3:**
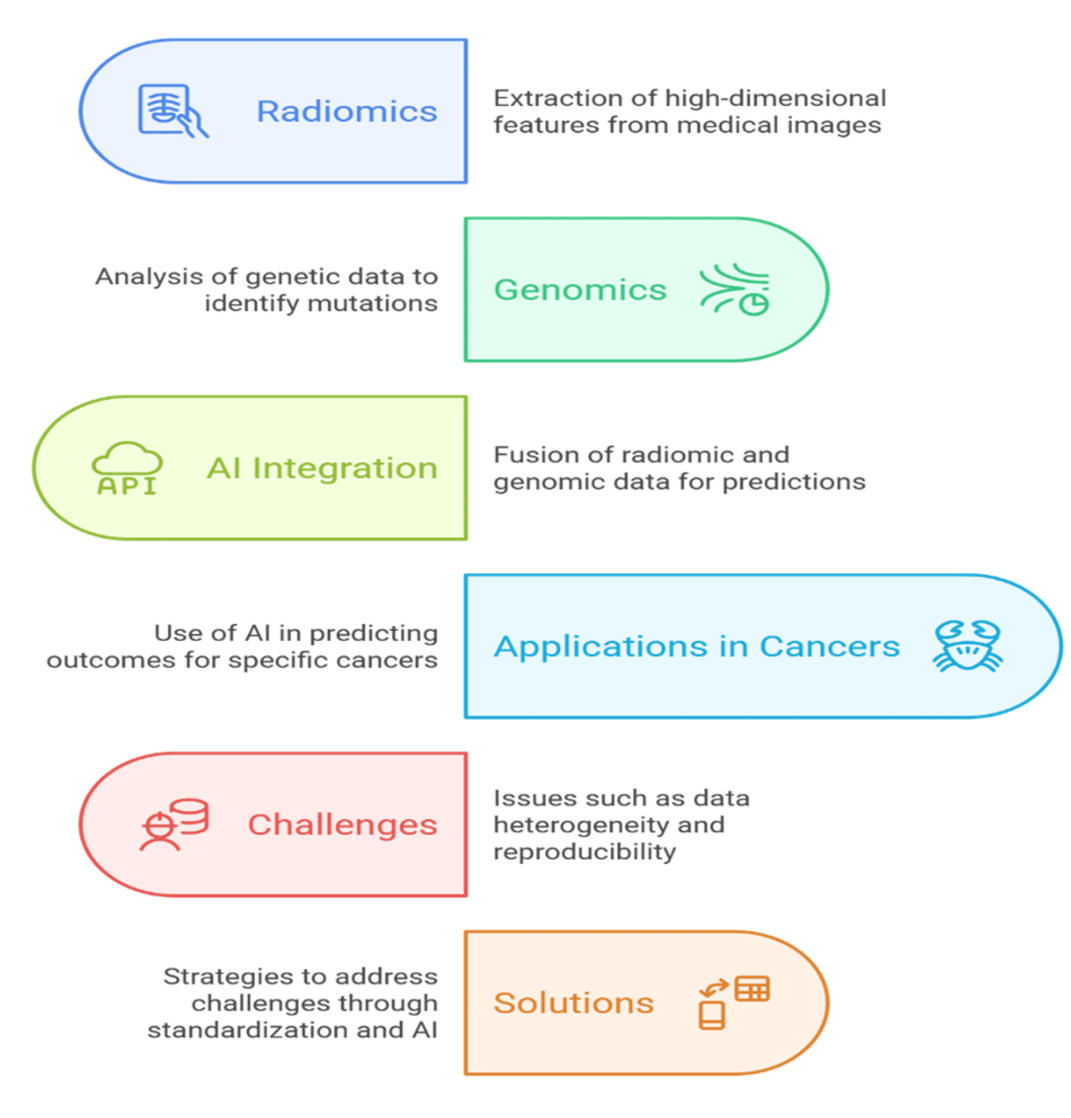
AI-enabled radiogenomics for predictive modeling in personalized oncology AI: artificial intelligence Image credit: Shubham Gupta

Nevertheless, challenges remain in ensuring data harmonization across institutions, which could be addressed through initiatives such as the adoption of standard imaging protocols, the establishment of centralized data repositories, and the implementation of federated learning models that allow data sharing without compromising patient privacy [46]. Different imaging acquisition protocols, segmentation approaches, and feature extraction pipelines make a difference in reproducibility [6]. To deal with this, initiatives such as the Image Biomarker Standardisation Initiative (IBSI) and other collaborative models are standardizing radiomic pipelines [47]. The multimodal data fusion is also currently performed on advanced AI architectures, including attention-based transformers and graph neural networks, which capture the interaction between imaging and molecular data [17]. Explainability is also critical. The Shapley Additive Explanations (SHAP) values and feature attribution maps, as well as attention weights, can be used to visualize the contribution of integrated features to the predictions, which is helpful in clinical trust and regulatory approval [48]. With the growing scope of federated learning and increasing datasets, the capacity of AI to combine radiomics and genomics will become the key to the development of predictive models that can be used to match diagnosis and therapy in real time. Figure 3 represents how AI integrates radiomic and genomic data to enable cancer-specific prediction, diagnosis, and therapy personalization.

Workflow optimization and radiologist-AI collaboration

Beyond detection and classification, AI is increasingly recognized for its ability to streamline and optimize radiological workflows [49]. One of the most impactful implementations is in triage systems, where AI rapidly reviews incoming studies and flags urgent findings such as intracranial hemorrhages or pulmonary embolisms, thereby accelerating clinical response in emergency settings [41]. In high-volume imaging environments like mammography or chest radiography, AI contributes to workload management by identifying likely normal studies for expedited review or omission, thus conserving radiologist attention for complex or ambiguous cases [42]. Evidence suggests that such human-in-the-loop systems can reduce reading volume by nearly 30% without compromising diagnostic sensitivity [43].

AI’s utility extends into clinical decision support, where structured reporting tools aid in the categorization of liver lesions, musculoskeletal abnormalities, and risk profiling in lung nodule screening [44]. These systems enhance consistency and help reduce interobserver variability. Additionally, AI-enabled platforms offer natural language processing (NLP) functionalities that convert radiologic findings into structured reports, reducing documentation time while maintaining clarity [45]. Tools like Nuance PowerScribe One exemplify this advancement. In parallel, transparency tools such as heatmaps, case-based reasoning modules, and confidence scores are being deployed to support interpretability and clinician trust [46]. AI also serves an important role in training environments, providing annotated feedback that accelerates learning and standardizes diagnostic performance among junior radiologists [37].

A recent evolution in this domain is the emergence of agentic AI, AI systems capable of autonomously initiating, monitoring, and adapting tasks within radiological workflows [5,11,35]. Unlike traditional passive algorithms that await user prompts, agentic AI can dynamically orchestrate imaging pipelines, prioritize cases based on urgency, and self-correct through reinforcement feedback loops [2]. In radiology, such agentic systems may automatically flag protocol deviations, suggest optimal imaging parameters, or initiate cross-modality comparisons without human command [38]. This paradigm shift represents a move from assistive to proactive AI, fostering continuous workflow optimization and adaptive collaboration between human experts and intelligent systems. As a result, radiologists transition from passive users to supervisory partners overseeing AI-driven, context-aware diagnostic ecosystems.

Despite these benefits, integration hurdles remain. Interoperability with existing PACS/radiology information system (RIS) systems, with upfront implementation costs ranging from $200,000 to over $1 million depending on the system's complexity and scale, and medicolegal ambiguities continue to slow widespread adoption [25]. However, early adopters report measurable gains in reporting speed, diagnostic accuracy, and team coordination, positioning AI as a key collaborator in the future of radiology [50]. Table 3 showcases the role of AI-integrated radiogenomics in personalized oncology, highlighting how radiomics, genomics, and advanced AI methods converge to support non-invasive prediction, treatment selection, and individualized care.

**Table 3:** Applications of AI in radiological workflow optimization AI: artificial intelligence; DSS: decision support system; LI-RADS: Liver Imaging Reporting and Data System; NLP: natural language processing; PACS/RIS: picture archiving and communication system/radiology information system; LLM: large language model

Workflow area	AI application	Clinical impact	Tools	References
Triage & Prioritization	Automated flagging of critical cases (e.g., hemorrhage and embolism)	Faster emergency response, improved patient safety	AI triage algorithms integrated into worklists	Hou et al. [41]
Fatigue Reduction	Pre-screening of normal or negative studies	Reduced radiologist workload by ~30% without compromising sensitivity	Human-in-the-loop systems, screening AI	Harmon et al. [20]
Risk Stratification	AI-based malignancy scoring during screening workflows	Improves diagnostic focus, supports decision-making	Virtual Nodule Clinic (Optellum)	Sadoughi et al. [12]
Structured Reporting	Suggesting standardized report elements based on lesion characteristics	Increased reporting consistency and decreased interobserver variability	AI-enabled DSS (e.g., for LI-RADS in liver imaging)	Santos et al. [23]
Musculoskeletal Imaging	Auto-assignment of clinical scores for fracture risk or lesion classification	Supports standardization in orthopedic diagnostics	ACR scoring AI tools, NLP models	Mun et al. [4]
Radiology Reporting	NLP for summarizing findings into structured reports	Decreased reporting time and increased clarity	Nuance PowerScribe One, AI-generated summaries	Dangi et al. [10]
Protocol Optimization	Automates sequence selection and ensures image quality	Ensures relevant scans; flags motion artifacts or poor contrast timing	Quality control algorithms	Rompianesi et al. [21]
Transparency Tools	Visual explanations, confidence scoring, and case-based learning	Builds trust and interpretability of AI outputs	Aidoc, TeraRecon, heatmaps, confidence intervals	Kumar et al. [30]
Education & Training	Annotated AI feedback for learners	Faster learning, decreased diagnostic variability among junior readers	AI annotation tools, training modules	Najjar [5]
Implementation Challenges	Interoperability, cost, and medicolegal ambiguity	Slows adoption; requires system-wide integration	PACS/RIS integration barriers	Kim and MacKinnon [32]
Generative AI (LLMs)	Plain-language report generation, patient-friendly summaries, and automated documentation	Improves communication between radiologists, clinicians, and patients; reduces reporting burden	ChatGPT, GPT-4-based LLMs for radiology report simplification	Sarangi [51]; Sarangi et al. [52]

Ethical, regulatory, and implementation challenges

Along with the introduction of AI in radiology processes, there is a substantial ethical, regulatory, and operational challenge. One of them is bias in AI models because of the lack of representativeness of training data. As an example, skin lesion models trained on lighter skin types do not perform well in darker-skinned populations. On the same note, models of chest X-rays can underdiagnose pneumonia among children or pregnant individuals in case the subgroups are underrepresented [46]. The other important issue is black-box DL. Radiologists and referring physicians need to know not only what the AI predicts but also why. Visual or statistical explanations of model outputs are now provided by XAI methods like LIME, SHAP, and Grad-CAM, although adoption is inconsistent [37].

The privacy of the data is the most important thing, considering that AI needs substantial amounts of data. The regulatory systems, such as General Data Protection Regulation (GDPR) and Health Insurance Portability and Accountability Act (HIPAA), require rigid data processing, and the newer technology, such as federated learning, permits the training of models on distributed data without centralizing data, which keeps patients confidential [47]. In India, integrating AI with the local PCPNDT (Pre-Conception and Pre-Natal Diagnostic Techniques) Act would require ensuring that all data usage, especially related to prenatal imaging and diagnostics, complies with the strict guidelines for the prevention of sex determination and related misuse. Federated learning could be particularly useful here, as it allows data from different sources to be used for training without violating the act's regulations on data centralization and misuse [7]. Regulatory-wise, the agencies such as the FDA have guided in the context of Software as a Medical Device (SaMD). Several radiology AI products, e.g., Zebra Medical Vision and Viz.ai, have been cleared under 510(k) or approved under De Novo [48]. The gray areas, however, are the real-world monitoring, continuous learning updates, and post-market surveillance. Implementation barriers also persist. The hospitals require infrastructure developments to support the AI tools, such as graphics processing unit (GPU) clusters and secure RIS/PACS integration. Training of clinicians and AI literacy are also important, particularly in terms of assessing alerts, false positives, and confidence scores [49].

Another issue is the legal liability: who is to blame in case AI fails to make a diagnosis? Courts and regulatory bodies are still establishing precedent. Accordingly, the majority of deployments have human supervision in order to protect decision-making. The moral law of justice requires that AI cannot widen the gap in care. Developers should perform an audit to eliminate bias, regulators should demand transparency, and hospitals should adopt equal distribution of resources, including access to AI tools and training, to ensure fairness in their deployment across diverse populations and healthcare settings. The key to the successful integration of AI into the care that does not negatively affect autonomy and safety is collaborative governance between radiologists, developers, ethicists, and patients [50].

Limitations and future recommendations

Although there has been a significant advancement, there are various limitations in the implementation of AI in radiology. The main issue here is that the performance of the model varies with the differences in imaging protocols, the type of scanners, and the population demographics of various healthcare systems. The AI technologies that show good results in the development environment cannot be successfully applied to real-life clinical environments. This is further worsened by the reliance on the large annotated datasets that are expensive to generate and also not evenly distributed throughout the regions. There is also a lack of standardization in image acquisition, labeling, and reporting that further makes model reproducibility and validation difficult. The design of clinical trials with AI tools is also not as well-developed, with the regulatory uncertainty slowing down the progress. Despite the existence of frameworks such as the FDA SaMD pilot, most AI systems change too quickly to be pursued through conventional approval channels.

To overcome these obstacles, in the future, we should focus on multi-institutional partnerships and open repositories (TCIA and the MIDRC) to develop diverse and high-quality datasets. The addition of XAI will be essential to increase the level of transparency and trust, particularly in situations that require high-stakes diagnosis. It is necessary to have seamless integration with PACS and hospital IT systems to facilitate clinical adoption. Post-deployment surveillance should be continuous to ensure model reliability and safety in practice. New methods like federated learning will be able to help with multicenter model improvement without affecting the privacy of patients. Lastly, well-organized education and training modules will be critical in equipping radiologists to engage in collaborative, AI-augmented decision-making and in developing responsible and evidence-based use of the technologies.

## Conclusions

The role of radiology in oncology is being transformed by AI, particularly ML and DL models, which can provide higher diagnostic accuracy, streamline workflows, and allow data-driven decision-making in oncology, including breast, lung, and prostate cancers, when properly implemented. With the aid of DL, radiomics, and radiogenomics, AI can perform as well as an expert on interpreting images, characterizing tumors, and modeling prognoses, and has transformative potential when it comes to early detection and personalized treatment. The integrated systems enable not only the identification of lesions but also molecular profiling, which is important in precision oncology. Even so, the practical application is still hampered by the problems of generalizability, bias of the dataset, ethical ambiguity, and lack of integration in clinical practice. These shortcomings render the necessity of multicenter validation, federated learning models, and regulatory clarification. Ability to fit into the existing hospital infrastructure, along with clinician training and XAI tools, will be critical to clinical adoption. Notably, at the current stage of AI development, it should be used to supplement, rather than substitute, the radiologist's expertise. This approach enhances diagnostic certainty and effectiveness while maintaining human oversight. The evidence to date highlights the ability of AI to transform oncologic imaging into a predictive and personalized platform. In the future, long-term interdisciplinary cooperation and intensive post-market monitoring are necessary to make sure that AI technologies are safe, equitable, and strongly validated. This kind of strategy will make it so that the AI integration in cancer radiology will not only be possible technologically but also be a necessity in clinics.
